# Overview of Median Arcuate Ligament Syndrome: A Narrative Review

**DOI:** 10.7759/cureus.46675

**Published:** 2023-10-08

**Authors:** Will Upshaw, John Richey, Gurjot Ravi, Adrian Chen, Noah J Spillers, Shahab Ahmadzadeh, Giustino Varrassi, Sahar Shekoohi, Alan D Kaye

**Affiliations:** 1 Medicine, Louisiana State University Health Sciences Center, Shreveport, USA; 2 Medicine, Ross University School of Medicine, Shreveport, USA; 3 Anesthesiology, Louisiana State University Health Sciences Center, Shreveport, USA; 4 Pain Medicine, Paolo Procacci Foundation, Rome, ITA

**Keywords:** ischemia, compression, celiac artery, diaphragm, median arcuate ligament syndrome

## Abstract

Median arcuate ligament syndrome (MALS) is a rare disorder caused primarily by compression of the celiac trunk by the median arcuate ligament (MAL). This disorder typically results in patients presenting with bloating, weight loss, nausea, vomiting, and abdominal pain. The MALS diagnosis is one of exclusion, as the disorder has no specific diagnostic criteria. Imaging modalities are often utilized to assist in making the diagnosis, such as ultrasound, computed tomography angiography (CTA), and magnetic resonance angiography (MRA). These imaging modalities typically reveal a stenosed celiac artery with post-stenotic dilation in patients. This disorder is usually treated by dividing the MAL, thus relieving the compression of the celiac artery. The surgery may be done through either an open approach or a minimally invasive approach, which can be either laparoscopic or robot-assisted. Most patients respond well to this treatment, though certain factors that predict a poorer response to treatment include elderly age, a history of alcohol abuse, and psychiatric illness.

## Introduction and background

Median arcuate ligament syndrome (MALS) is a rare disorder that affects approximately 2 per 100,000 people [1]. It is believed to be caused by the constriction of the celiac artery and the celiac plexus by the median arcuate ligament (MAL) [2]. The MAL serves to connect the right and left crura of the diaphragm and is typically found at the level of either the twelfth thoracic or the first lumbar vertebrae, where it forms the anterior border of the aortic hiatus [3]. Immediately below the MAL is the celiac artery, an unpaired artery branching off the abdominal aorta. This artery has numerous branches that arise from it, which are crucial in supplying blood to the spleen and the structures derived from the embryological foregut [4]. Additionally, located next to the celiac artery is the celiac plexus. This plexus is formed from sympathetic and parasympathetic nerve fibers that transmit information to and from structures derived from the embryological foregut and midgut [5].

When the MAL is found inferior to its typical insertion on the spine or when the celiac artery is superior to where it typically branches from the thoracic aorta, MALS may develop. While the reason for this anatomical malformation is unknown, it is believed that both hereditary and environmental factors likely play a role in the development of MALS [6]. Due to this abnormal anatomical structure, the celiac artery and the celiac plexus are compressed by the MAL. This compression may result in hyperplasia of the intimal wall of the celiac artery, thus causing stenosis of its lumen and ultimately resulting in ischemia of the abdominal organs [2]. Additionally, there may be issues caused by abnormalities in the transmission of neural impulses from the abdominal organs due to the pressure exerted on the celiac plexus by the MAL [3]. As a result of these pathologic changes, patients may experience certain symptoms such as nausea, vomiting, postprandial pain, and weight loss [7]. The symptoms are typically more severe upon expiration due to the diaphragm moving caudally, resulting in greater celiac trunk compression by the MAL [3,8].

The diagnosis of MALS is a diagnosis of exclusion. Various imaging modalities may be utilized to rule out other diseases, such as CT and ultrasound [9]. If the patient does suffer from MALS, imaging will reveal a stenosed celiac artery with arterial dilation distal to the stenosed region. Upon diagnosis, treatment is generally surgical, which involves the excision of the MAL, resulting in decompression of the celiac trunk and plexus [10].

As MALS is such a rare disease, it may be difficult to diagnose in patients. The purpose of this article is to shed light on this rare syndrome, as this will hopefully aid clinicians in the recognition of this disorder. Thus, in this article, we discuss several factors related to MALS, such as its treatment, symptoms, epidemiology, and the most effective imaging methods to aid in the diagnosis of this disease.

## Review

Methods

This is a narrative review. The sources for this review are PubMed, Google Scholar, Medline, and ScienceDirect. These databases were searched using the following keywords: median arcuate ligament syndrome, diaphragm, celiac artery, compression, and ischemia. Sources were accessed between May 2023 and July 2023.

Presentation

The prevalence of MALS is uncertain, primarily because its clinical manifestations can vary significantly, with many asymptomatic cases only being discovered after patients undergo imaging with a CT scan. That said, it is estimated that 13% to 50% of the population has some degree of compression of the celiac artery by the MAL [11]. The typical onset of symptoms is between 30 and 40 years of age; however, there are reported cases in pediatric patients. Additionally, females are four times more likely to develop the condition than males, and women with a thin body frame are at an even greater risk of developing MALS [2]. Other risk factors for MALS include smoking, hypertension, hyperlipidemia, malnutrition, and prior abdominal surgeries [12].

Mental disorders such as anxiety disorders, depression, panic disorder, and post-traumatic stress disorder are common comorbidities that present alongside MALS in many cases [13]. These disorders are especially common in pediatric patients who suffer from MALS. This was shown in a study conducted by Stiles-Shields et al., where over half of the pediatric patients evaluated for MALS had a comorbid psychological condition [14]. Other disorders characterized by physical abnormalities are present at higher rates in patients with MALS than in the general population, such as visceral vascular abnormalities, Ehlers-Danlos syndrome, and postural orthostatic tachycardia syndrome [15].

Commonly reported symptoms of MALS include epigastric pain that is intensified after eating, severe abdominal pain, weight loss, diarrhea, and vomiting [2]. Abnormal sounds, referred to as abdominal bruits, may also be detected in up to 35% of MALS patients upon clinical examination [16]. The symptoms of MALS are believed to be due to the abnormally low insertion of the MAL, which occurs because of errors in embryological development. This low insertion of the MAL results in compression of the celiac artery, causing a decrease in blood flow to the foregut. In addition, the celiac plexus is often compressed in patients with MALS, altering the neural control of digestive processes in these patients. This may cause these patients to have an abnormal gastric electrical rhythm and delayed gastric emptying, leading to their suffering from various digestive issues [2,17].

Many patients with MALS are asymptomatic due to the extensive collateral circulation supplying the foregut. The arteries that are part of this collateral circulation include the pancreaticoduodenal arcades, common hepatic artery, splenic artery, and dorsal pancreatic artery, among others. However, even if these patients do not experience the typical symptoms associated with MALS, the elevated flow of blood through these small collateral arteries significantly increases the risk of life-threatening aneurysm formation and rupture [18].

Diagnostic imaging

As stated earlier, compression of the celiac trunk by the MAL may lead to patients suffering from issues related to ischemia of the foregut organs. However, this ischemia due to the narrowing of the celiac artery caused by MAL compression is different from the narrowing of this vessel due to atherosclerosis, even though both cause turbulent blood flow through the celiac artery. The hook sign helps to differentiate between external compression of the artery by the MAL and pathological narrowing of the vessel due to atherosclerosis [19]. This sign reveals an upward bend of the celiac artery due to external compression. In patients with the diagnosis of MALS, the upward deflection of the celiac artery determines the degree of stenosis. Additionally, patients with a diagnosis of MALS have a much larger and more severe degree of deflection than those without this condition [20]. Thus, the degree of deflection of the celiac artery toward the aorta should be measured in patients who are suspected of having MALS as a way of determining the severity of this condition [21].

Imaging plays a key role in the diagnosis of MALS. There are multiple modalities to aid in this diagnosis, though some have proven to be more useful than others. The imaging modalities that may be used include computed tomography angiography (CTA), doppler ultrasound, and magnetic resonance angiography (MRA). Each of the modalities listed may be useful in determining the degree of stenosis in the celiac artery. However, while the use of imaging contributes to the conclusion of most diagnoses of MALS, imaging techniques should be used in conjunction with clinical symptoms to determine the severity of the disease. Additionally, the presence of the hook sign should not be the only factor examined on imaging, as the degree of the angle created by the deflection of the celiac artery should also be taken into consideration in these patients [20]. Phases of breathing are important to consider when evaluating patients for MALS, as the severity of stenosis is affected by this factor. As MALS is a product of external compression of the celiac artery by the MAL, during expiration, compression is worsened due to the caudal movement of the diaphragm [22].

**Figure 1:**
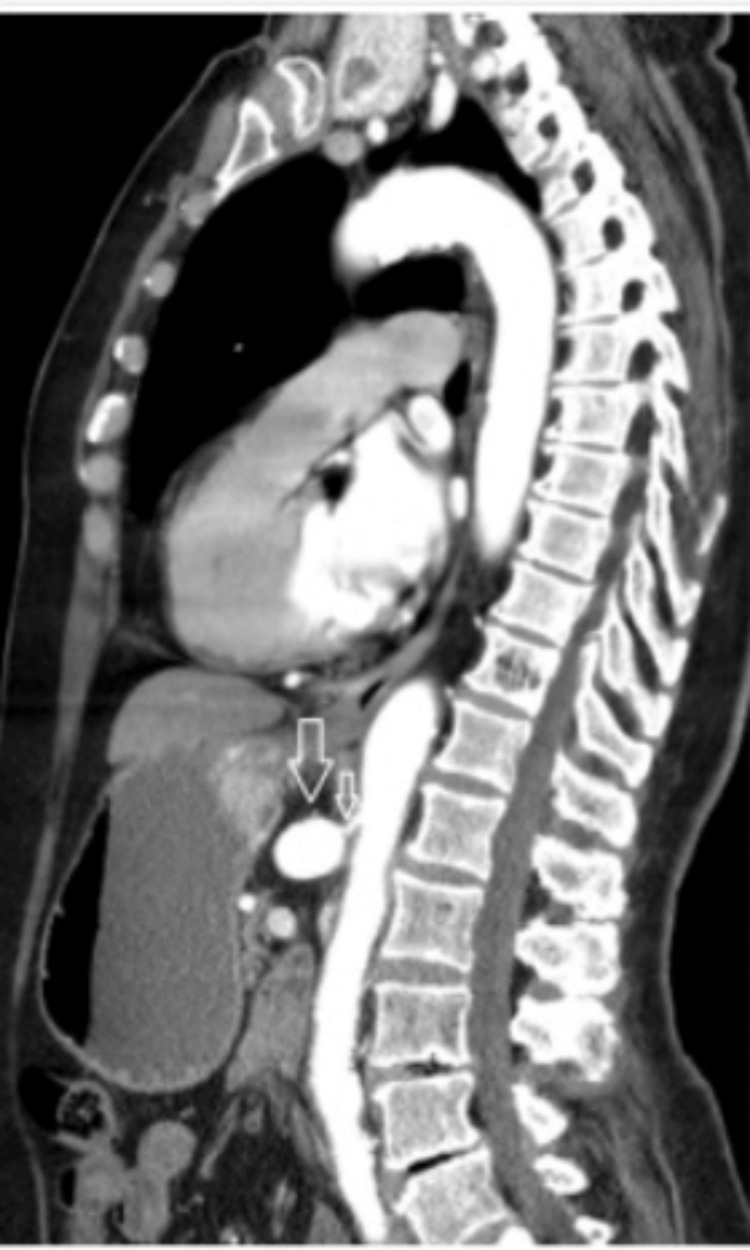
Sagittal CT image showing stenosis of the celiac artery in a patient with MALS MALS: Median arcuate ligament syndrome

Evaluation of MALS in patients using CTA works by determining the degree of stenosis via the arterial phase during expiration and the venous phase during inspiration. Multiple scanners are used to create a multiplanar construction that can be seen as a three-dimensional image, as shown in Figure 1 [23]. This helps to determine whether there is external compression of the celiac trunk due to the MAL. The MRA also works similarly in that it uses the phase of breathing to help compute an image of the celiac trunk to gauge the degree of stenosis [24]. Taking the phases of breathing into account is important, as it has been shown that both CTA and MRA produced more sensitive and specific diagnostic results when the use of the breathing phases was employed [24]. Multiple factors contribute to the use of one modality over another, though CTA has proven especially useful in evaluating patients for MALS. Some of the specific benefits of CTA are that it can be reproduced easily and has a lower interobserver variation [22]. Further benefits of CTA are that it is non-invasive, produces a multiplanar reconstruction of the arterial planes with detailed resolution, and has rapid image procurement [25]. Unfortunately, CTA does come with some downsides. These disadvantages to using CTA are contrast-induced nephropathy and radiation exposure, resulting in restrictions as to which patients can undergo this imaging technique [22].

**Figure 2:**
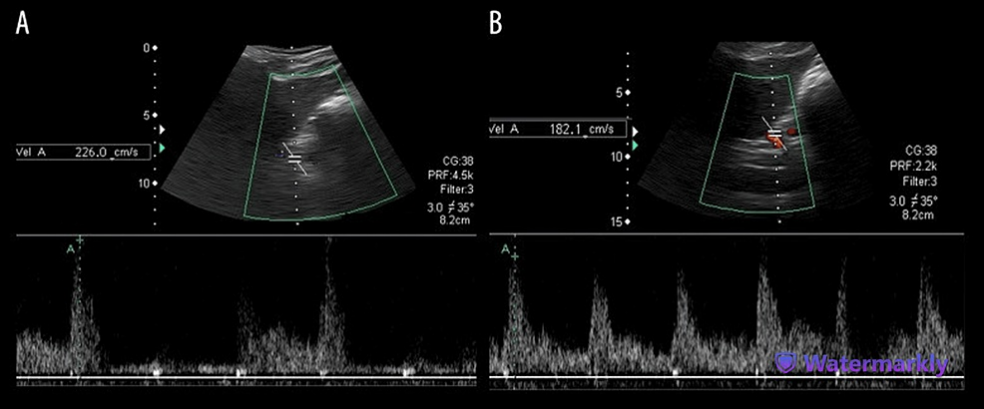
Duplex Doppler ultrasound showing the stenosed celiac artery worsening when a patient with MALS undergoes forced expiration MALS: Median arcuate ligament syndrome

Doppler ultrasound can also be used to evaluate patients for MALS, as it can detect the angle of deflection of the celiac artery as well as the inspiratory and resting arterial flow velocities through this vessel [23]. Using the Doppler ultrasound, the presence of the hook sign, which is shown in Figure 2, may also be determined, making this a good preoperative image construction technique. However, with its kinetic nature, measuring during breathing phases is necessary. Even with these complications, it is still very reliable, with 75% and 89% sensitivity and specificity, respectively, when used in making a diagnosis of MALS [25]. Another benefit of Doppler ultrasound is that it avoids the use of radiation and contrast, making it a safe imaging modality for patients. Although the signs and degree of deflection can be seen with Doppler ultrasound, MRAs are superior to Doppler ultrasound in certain circumstances, such as in the diagnosis and preoperative planning of MALS in pediatric populations. Additionally, the effectiveness of Doppler ultrasound may be reduced by certain variables, such as the amount of air in the gut, which can obstruct image capture with this modality [26].

Although all imaging modalities have their own respective advantages and disadvantages, they are still all used today to detect the presence of positive MALS findings with great success. When determining the best technique to assess for MALS in patients, a multifactorial approach should be considered. Additional factors that should be considered when making a diagnosis of MALS are patient history, age, and symptoms.

Treatment

Since MALS is caused by compression of the celiac artery by the MAL, treatment for this disorder is centered around decompression of the celiac artery. This is performed so that blood flow to the upper abdomen can be re-established, thus preventing ischemic issues from continuing [11]. Decompression of the celiac artery is achieved through surgical intervention as opposed to other nonoperative treatment methods. This can be performed through open or minimally invasive approaches, which include either laparoscopic or robot-assisted techniques [27]. Surgical intervention aims to carry out the complete division of overlying tissue from the diaphragmatic crura, which forms the MAL, along with potential neurolysis of the celiac plexus nerve fibers to aid in pain relief [28]. An intraoperative duplex ultrasonography can be used, or a conformational change of the celiac artery can be assessed to determine adequate release of celiac artery compression by confirming there is minimal celiac artery elevation velocity and no change with deep respiration [11].

Decompression of the celiac artery was traditionally accomplished by using an open approach to surgery. However, there has been a shift in recent years to performing this surgery utilizing a minimally invasive approach, with this involving either laparoscopic or robot-assisted techniques [2]. Although both open and minimally invasive techniques have been shown to have comparable outcomes, the advantages of the laparoscopic technique are that there is generally decreased postoperative pain, a shorter hospital stay, faster recovery times, and improved cosmetic outcomes [29].

Even though surgical decompression of the MAL is extremely effective in relieving patients of their symptoms, there are still some complications that have been reported following the procedure. Complications that can occur with open surgery include postoperative thrombosed bypass graft with stroke, gastroesophageal reflux disease, pancreatitis, hemothorax, and splenic infarction. Complications that occur with a laparoscopic approach include celiac artery bleeding, pneumothorax, aortic puncture, phrenic artery laceration, and gastric artery bleeding [10]. Additional drawbacks with minimally invasive techniques include increased difficulty managing hemorrhage, which may be caused by an injury to the aorta or one of its branches. This may ultimately require conversion to open surgery if the vascular injury is severe enough so that bleeding can be effectively managed [30]. Robot-assisted techniques may also be used with this approach, offering improved surgical precision along with more stability and an increased ability to perform intricate maneuvers in confined spaces. Robotics also allows for enhanced three-dimensional perception, which contributes to improved control and dexterity, permitting better navigation around the celiac artery when attempting to divide the MAL [31]. However, there are drawbacks to utilizing a robot-assisted approach: it is costly, requires large port incisions to be made, and has poor haptic feedback for the surgeon [10].

Additional procedures such as revascularization of the celiac artery by stenting, celiac artery bypass surgery, and celiac ablation can be done if stenosis persists after MAL decompression [28]. Since a delay in the celiac artery revascularization procedure is unlikely to adversely affect the outcome, surgery to release the constraining MAL is the primary treatment, followed by celiac artery revascularization if symptoms persist. However, surgery involving ligament release along with revascularization has been shown to significantly increase symptom relief long-term, though revascularization alone is largely avoided due to clinical failure and recurrent stenosis [31]. Additionally, the hybrid treatment of combined laparoscopy and percutaneous angioplasty with stent implantation appears to result in long-term resolution of symptoms by restoring normal function and minimizing the risk of restenosis [32]. Similarly, celiac ganglion ablation following ligament release may lead to long-term symptom improvement [28].

Prognosis

Data regarding the outcomes of patients who received treatment for MALS is lacking and has yielded somewhat conflicting results. There are small sample studies and reviews that report on the results of treatment for MALS. These studies have shown that surgical decompression appears to provide short-term symptom relief in patients, though data is lacking for the long-term reoccurrence of symptoms in these patients [2]. One review found that in 35 studies involving a total of 691 patients, 75% to 100% of patients reported a clear reduction in symptoms after undergoing surgery [13]. In another review of studies, 85% of MALS patients who underwent surgical intervention reported immediate symptom relief after surgery. Within this group of 400 patients, 279 patients received open surgery, with 78% reporting immediate relief, while 121 underwent laparoscopic division, with 96% reporting immediate relief [16]. A study by DeCarlo et al. compared outcomes between patients who underwent either open, laparoscopic, or robotic surgery for MALS. Their study showed that 49.3%, 62.4%, and 37.7% of patients had symptom relief at a three-year follow-up after undergoing open, laparoscopic, and robotic approaches to MALS surgery, respectively [33]. In a study by Shin et al. that compared laparoscopic and robotic surgery for MALS, 50 patients underwent either of the two approaches to surgery. Of the 24 patients receiving laparoscopic surgery, 76.9% reported symptom relief at a six-month follow-up, with a mean operation time of 86 minutes, a mean hospital stay of one day, and a mean blood loss of 9.8 ml. However, of the 26 patients who received robotic surgery, only 50% reported symptom relief at a six-month follow-up, with a mean operation time of 134 minutes, a mean hospital stay of one day, and a total blood loss of 13.0 ml [34]. In another study by Gerull et al. testing the effectiveness of robotic surgery for MALS, 74 patients received this procedure, with 90.3% reporting symptom relief at a one-year follow-up [35]. In a separate study by Chen et al. comparing open, laparoscopic, and robotic surgery for MALS, the cohort of 16 patients who underwent open surgery reported 62% symptom relief, while the laparoscopic cohort of 12 patients reported 20% symptom relief, and the robotic cohort of 17 patients reported 43% symptom relief, with all follow-ups being conducted one year after surgery [36]. Other perioperative variables from these studies, including operation time, hospital stay, and blood loss, are listed in Table 1.

**Table 1:** Outcome variables of 685 patients after surgery for MALS MALS: Median arcuate ligament syndrome

Reference	Number of Patients	Type of Surgery	Symptom Relief	Operation Time, minutes (mean)	Hospital Stay (mean)	Blood loss, milliliters (mean)
Decarlo et al. [33]	227	Open	43.9% (3 years)	179.1 +/- 78.7	5 Days	75.0 ml
Decarlo et al. [33]	235	Laparoscopic	62.4% (3 years)	107.5 +/- 41.8	2 Days	50.0 ml
Decarlo et al. [33]	54	Robotic	37.7% (3 years)	170.6 +/- 54.3	2 Days	40.0 ml
Shin et al. [34]	24	Laparoscopic	76.9 % (6 months)	86	1 Day	9.8 ml
Shin et al. [34]	26	Robotic	50% (6 months)	134	1 Day	13.0 ml
Gerull et al. [35]	74	Robotic	90.3% (1 year)	52.6 +/- 18.1	0.8 Days +/- .3	13.9 +/- 8.4 ml
Chen et al. [36]	16	Open	62% (1 year)	246 +/- 161	-	151 ml
Chen et al. [36]	12	Laparoscopic	20% (1 year)	204 +/- 53	-	100 ml
Chen et al. [36]	17	Robotic	43% (1 year)	141 +/- 69	-	15

## Conclusions

Median arcuate ligament syndrome is a rare disorder that is often difficult to recognize in patients who present with this condition. This disorder is believed to be caused primarily by compression of the celiac plexus and celiac artery by the MAL. When patients present with MALS, they typically have symptoms such as vomiting or nausea, bloating, weight loss, and severe abdominal pain. There are several imaging modalities, such as Doppler ultrasound, CTA, and MRA, that may be utilized by physicians to evaluate the celiac artery, thus aiding in the recognition of this disease. Treatment for this condition is surgical decompression of the celiac artery and celiac plexus through division of the MAL. This surgery may be done either through an open approach, a laparoscopic approach, or a robot-assisted approach. We hope that this review will aid physicians in making the diagnosis of this rare condition so that patients afflicted by MALS can begin treatment sooner to achieve better outcomes.
